# Impact of Infectious Disease Consultation on Clinical Management and Outcome of Patients with Bloodstream Infection: a Retrospective Cohort Study

**DOI:** 10.1038/s41598-017-13055-2

**Published:** 2017-10-10

**Authors:** Guangmin Tang, Liang Huang, Zhiyong Zong

**Affiliations:** 1 Center of Infectious Diseases, West China Hospital, Sichuan University, Chengdu, China; 2 Department of Infection Control, West China Hospital, Sichuan University, Chengdu, China

## Abstract

The impact of consultation by infectious diseases (ID) physicians on management and outcomes of patients has not been determined in China. We assembled a retrospective cohort of 995 consecutive adult cases with bloodstream infections (BSI) in a major teaching hospital in China. Survival analysis was performed with Cox regression and the Kaplan-Meier curves. Among the 995 patients with BSI, 421 (42.3%) received consultation by ID physicians and 574 (57.7%) did not. ID consultation led to a significant lower hazard of death (hazard ratio [HR], 0.575; *P* < 0.05) and more appropriate antimicrobial use (95.0% vs 67.6%, *P* < 0.05). ID consultation was a protective factor among patients with BSI due to Gram-positive (HR, 0.551; *P* < 0.05) or Gram-negative (HR, 0.331; *P* < 0.05) bacteria. Multiple ID consultation was a protective factor (HR, 0.51; *P* < 0.05), while single consultation was not. In conclusion, ID consultation led to significant lower risk for patients with BSI and improved management. Multiple rather than single ID consultations reduced the hazard of death.

## Introduction

The consultation by infectious disease (ID) physicians has been associated with improved clinical management for a variety of infections including urinary tract infections, osteomyelitis, fungaemia due to *Candida glabrata*, solid organ transplant recipients admitted for infections, bacteremia and bacterial endocarditis^1–8^. However, there were discrepancies in whether ID consultation led to better outcomes such as reduced mortality or not. Some studies found that ID consultation did not significantly decrease mortality after adjusting for multiple variables^2,3,9^, but some others reported more survivals associated with ID consultation^4–6,10–13^.

In China, few hospitals have infectious diseases (ID) physicians who specialized in ID other than viral hepatitis and tuberculosis^14^. Along with the national campaign for rational use of antimicrobial agents in clinical settings, the Ministry of Health, China, required all secondary or tertiary general hospitals to cultivate their ID physicians. However, the impact of the consultation of ID physicians on clinical management and outcomes of patients with ID has not been demonstrated in China. We therefore preformed a retrospective analysis on the impact of ID consultation on patients with bloodstream infections in a large teaching hospital in China. This study intended to confirm the hypothesis that ID consultation would be associated with better management and outcomes in patients with bloodstream infection regardless of the species of pathogens.

## Methods

### Study Design

We conducted a retrospective cohort study at West China Hospital of Sichuan University with 4,300 beds and about 210,000 admissions to the hospital in 2014. Consecutive inpatients were eligible for inclusion from 1 Jan 2014 to 31 Dec 2014. Patient records were reviewed if the patient had at least one positive blood culture but those with only one blood culture positive for common skin commensal microorganisms including coagulase-negative staphylococci, non-*diphtheriae Corynebacterium* spp., *Bacillus* spp., *Propionibacterium* spp., viridans group streptococci, *Aerococcus* spp., and *Micrococcus* spp.^15^ were excluded. Each admission was treated as an independent event and therefore some patients (n = 25) had been included in the study more than once. Only the first episode of bloodstream infection was included if the patient had repeated bloodstream infection during each hospitalization. Patients < 14 years of age were excluded from analysis as this population was referred to pediatricians rather than ID physicians at West China Hospital of Sichuan University.

This study was conducted in accordance with the amended Declaration of Helsinki and was approved by the Ethics Committee of West China Hospital with the informed consent being waived.

### Data Collection

Data were retrieved retrospectively from patients’ electronic medical records and were inputted into a self-designed case report form. Data collected included patient demographics, main underlying diseases, microbiological data, clinical management (receiving an echocardiogram, repeated blood culture, appropriate empiric antimicrobial therapy, appropriate definitive antimicrobial therapy and source control for infection foci), the length of stay (LOS) in hospital, ID consultation and clinical outcomes (death in the hospital or immediately after discharge). The right censoring data were the records of patients who discharged from hospital not because of diseases irreversible deterioration.

### Definition

For patient underlying diseases, immune suppression was defined as receiving corticosteroid at a dose of >10 mg prednisone or equivalent for at least 2 weeks, human immunodeficiency virus infection/AIDS, chemotherapy within last 6 weeks of the bloodstream infection onset, neutropenia within 72 hours of the bloodstream infection onset, or transplantation requiring immunosuppressive therapy^10^. Renal insufficiency was defined as a serum creatinine level >177 μmol/L within 24 hours of the bloodstream infection onset. Endocarditis was diagnosed using the modified Duke criteria^16^.

For the index blood stream infection, pathogens were divided into three categories, Gram-positive (G+) bacteria, Gram-negative (G−) bacteria and fungi. The infection site was determined by radiological, bacteriological or pathological investigations or solely by clinical suspicion. Organ/space infection foci included meningitis, mediastinitis, pneumonia, endocarditis, purulent arthritis, osteomyelitis, deep-seated abscess and foreign-body infections^13^. Source control for infection foci included removal of intravascular devices, removal of catheters/stents if the patient had bloodstream infection associated with catheters or secondary to urinary tract infections, surgical/interventional drainage and cardiovascular surgery.

Appropriate antimicrobial therapy was defined as the regimen containing any antibiotic that was active *in vitro* against the recovered pathogen and was given by an appropriate route and in appropriate dosage consistent with current medical standards, such as The Sanford Guide to Antimicrobial Therapy^17^ and the national guidance in China^18^, after *in vitro* susceptibility report was available. Species identification and antimicrobial susceptibility tests of the pathogens were determined using the Vitek II automated microbiology system (bioMerieux, Lyon, France) following the breakpoints of the Clinical and Laboratory Standards Institute (CLSI) guideline^19^.

According to our Chinese customs, patients usually have a strong desire to pass away at home and therefore many patients would choose to give up treatment and discharge from hospitals when they do not respond to the treatment and feel that they would die soon. We regarded the patients who did not respond to the treatment and would die in hospital, which were judged by the consensus of two physicians in blind, but chose to discharge as predicted death. The date of discharge of patients with predicted death was considered as an outcome measure, which was equal to death, in the analysis.

### ID Consultation

For bloodstream infection cases, consultation by ID physicians who were not only specialized in treating viral hepatitis and tuberculosis but also in managing bacterial and fungal infections was available but optional at West China Hospital of Sichuan University. There were 20 such ID physicians available for consultation in the hospital. In addition, there was an ID ward with 88 beds for managing all kind of ID. Based on the published studies^13,20^, telephone ID consultation is inferior to bedside ID consultation. Indeed, telephone consultations are not allowed in our hospital and therefore in this study, ID consultation referred to a bedside consultation or having an ID physician as the most responsible physician (i.e. patients admitted to the ID ward).

### Outcomes

Primary outcome was mortality status at the time of hospital discharge. Patients with terminal diseases and at immediate death risk after hospital discharge were also classified as dying in the hospital.

### Statistical analysis

The COX models were used to evaluate hazard ratio (HR) values of cumulative effect of ID consultations and each covariate. HR values refer to death or predicted death. Relevant covariates were selected in a stepwise (PE = 0.05 and PR = 0.1) manner and the final model was also vetted by judgment of clinical significance. To test the assumption of proportional hazard in COX regressions, covariates with potential interaction with time from the clinical viewpoint were fitted as time-varying predictors. The subgroup COX models were established to identify whether the impact of ID consultation on outcome was different for Gram-positive bacteria, Gram-negative bacteria and fungi infection, using the identical approach as described above. We used single ID consultation (IDC) group/multi-IDC group/no ID consultation (NIDC) group or IDC/NIDC to group patients who had different IDC status with the NIDC group as the reference. The two models were used to evaluate if the impact of IDC was mainly from single IDC or multi-IDC. The COX regression included clinically significant covariates (Table 1) including sex, age, ward, pathogen, main underlying diseases, infection foci, central line catheterization, infective endocarditis, granulocytopenia, malignance, hypotensive shock (within 24 h of blood culture collection), dialysis, immune suppression, parenteral nutrition, renal insufficiency (within 24 h of blood culture collection), mechanical ventilation (within 7 d of blood culture collection), any appropriate definitive and empirical antimicrobial therapy, IDC, source control for infection foci and length of stay (LOS) after blood culture collection. The Kaplan-Meier (KM) method and the log-rank test were used to compare the survival distributions between groups. HR values were reported with 95% confidence intervals (CIs) and all tests were two-sided with a *P* < 0.05 significance level. The categorical values were tested by Fisher’s exact test and continuous variables (e.g. age) were compared using Wilcoxon rank-sum test, both of which were applied to data shown in Tables 1 and 2. All analyses were performed using the Stata/SE 12.0 statistical software (StataCorp LLC, College Station, Texas, USA).

**Table 1:** Baseline and clinical characteristics of the patients^a^.

Characteristics	All N = 995	IDC N = 421	NIDC N = 574	*P* value
Age (median)	55	52.4	55.5	0.005
Male	619	247	372	0.055
Ward				
ICU	180	38	142	0.000
Internal Medicine (except Hematology and Oncology)	246	116	130	0.000
Surgical	321	151	170	0.023
Emergence	138	77	61	0.001
Hematology	55	2	53	0.000
Oncology	48	34	14	0.000
Dermatology	3	2	1	0.577
Ophthalmology	4	1	3	0.642
Main underlying disease				
Action inconvenience, e.g. paralysis	79	35	44	0.723
Chronic hepatic and biliary system disease	247	92	155	0.064
Chronic heart disease	37	19	18	0.309
Chronic pulmonary disease	34	3	31	<0.001
Hematologic neoplasm	69	11	58	<0.001
Urinary tract diseases except chronic renal insufficiency	44	24	20	0.118
Vascular diseases e.g. aortic dissection	5	4	1	0.169
Chronic renal insufficiency	72	25	47	0.215
Skin diseases	4	3	1	0.317
Connective tissue disease	12	4	8	0.574
Diabetes	89	46	43	0.072
Burn or electric injury	13	4	9	0.574
Hypotensive shock (within 24 h)	209	68	141	0.001
Renal insufficiency (within 24 h)	116	33	83	0.001
Mechanical ventilation with 7 d	237	68	169	0.000
Dialysis	80	19	61	0.000
Malignance	289	125	164	0.724
Immune suppression	164	65	99	0.489
Granulocytopenia	76	16	60	0.000
Parenteral nutrition	177	71	106	0.000
Deep vein catheterization	420	143	277	0.000
Complicated by infective endocarditis	21	10	11	0.659
Pathogens				
Gram-positive bacteria	231	106	125	0.224
*S. aureus*	93	56	37	0.000
*E. faecium*	57	16	41	0.027
Gram-negative bacteria	677	279	398	0.335
*Enterobacter cloacae*	43	23	20	0.155
*E. coli*	326	144	182	0.413
*K. pneumoniae*	137	61	76	0.578
*Pseudomonas aeruginosa*	35	17	18	0.488
*Acinetobacter* spp.	61	16	45	0.011
Fungi	87	36	51	0.910
*Candida albicans*	26	8	18	0.315
other *Candida* spp.	50	21	29	1.000
Organ/space foci	622	260	362	0.691

^a^Abbreviations: IDC, infectious disease consultation; NIDC, non-infectious disease consultation.

**Table 2:** Managements and outcomes of patients with bloodstream infection^a^.

	All patients	IDC N = 421	NIDC N = 574	*P* value
Management				
Echocardiography	436	205	231	0.008
Repeated blood culture	657	310	347	<0.001
Source control for infection foci	477	185	292	0.034
Appropriate definitive antimicrobial therapy	903	400	503	<0.001
Appropriate empirical antimicrobial therapy	712	327	385	<0.001
Outcome				
LOS	15	18	14	<0.001
Mortality rate	17.2	13.3	20.0	0.006

^a^Abbreviations: IDC, infectious disease consultation; NIDC, non-infectious disease consultation. LOS, length of stay.

## Results

### General cohort and ID consultation

A total of 995 patients with bloodstream infection were eligible for the study, including 421 (42.3%) in the IDC group and 574 (57.7%) in the NIDC group. ID consultation coverage for patients with bloodstream infection was 42.3%. Within the IDC group, 363 (86.2%) patients had at least one ID consultation and 58 (13.8%) patients had an ID physician as the most responsible physician. The 995 patients aged from 14 to 102 years old including 619 male (62.2%) and 376 female (37.8%). The five most common pathogens of bloodstream infection were *Escherichia coli* (n = 326), *Klebsiella pneumoniae* (n = 137), *S. aureus* (n = 93), *Acinetobacter* spp. (n = 61) and *Enterococcus faecium* (n = 57). The baseline and clinical characteristics of the patients were outlined in Table 1.

### Clinical management and outcomes

Compared to patients of the NIDC group, IDC patients were more likely to receive an echocardiogram, a repeated blood culture, appropriate definitive antimicrobial therapy and source control for infection foci (Table 2). Among the 995 patients with bloodstream infection, 171 (17.2%, 171/995) either died (n = 107) or were predicted death (n = 64) at discharge including 56 (13.3%, 56/421; 37 died and 19 predicted death) in the IDC and 115 (20.0%, 115/574; 70 died and 45 predicted death) in the NIDC group. The proportion of predicted death in patients who either died or were predicted death was similar between the two groups (19/56, 33.9% in the IDC group vs 45/115, 39.1% in the NIDC group, *P* > 0.05).

For all patients, the median LOS after blood culture collection was 15 days: 18 days for IDC patients and 14 days for NIDC patients. For patients who survived in hospital, the median LOS after blood culture collection was 16 days: 18 days for IDC patients and 14 days for NIDC patients but a prolonged LOS may not reflect the actual benefit when compared to those died. We therefore used COX modeling to evaluate the impact of multiple factors on the hazard of death.

### Two-group of IDC COX modeling

Based on the COX modeling results, risk factors for the hazard of death were older age, accompanied with shock, renal insufficiency or mechanical ventilation and being admitted to Hematology Department or Oncology Department. In contrast, protective factors against the hazard of death included ID consultation, source control for infection foci and appropriate definitive antimicrobial therapy as shown in Table 3. HR value of the IDC group was 0.575 (*P* < 0.05), indicating significant advantages in survival over the NIDC group.

**Table 3:** HR values (the IDC group vs the NIDC group) generated from the COX model^a,b^.

Variables	HR	*P* value	95% CI
Age	1.020	<0.001	1.011–1.029
Hematology department	4.311	0.002	1.230–2.507
Oncology department	3.624	<0.001	1.771–7.419
ID consultation	0.575	0.002	0.404–0.819
Appropriate definitive antimicrobial therapy	0.560	0.017	0.348–0.902
Hypotensive shock	2.601	<0.001	1.817–3.722
Mechanical ventilation within 7 d	1.756	0.002	1.230–2.507
Source control for infection foci	0.486	<0.001	0.346–0.681
Renal insufficiency within 24 h	2.044	<0.001	1.382–3.024

^a^HR values refer to death or predicted death.

^b^Abbreviations: HR, hazard ration; IDC, infectious disease consultation; NIDC, non-infectious disease consultation.

**Figure 1:**
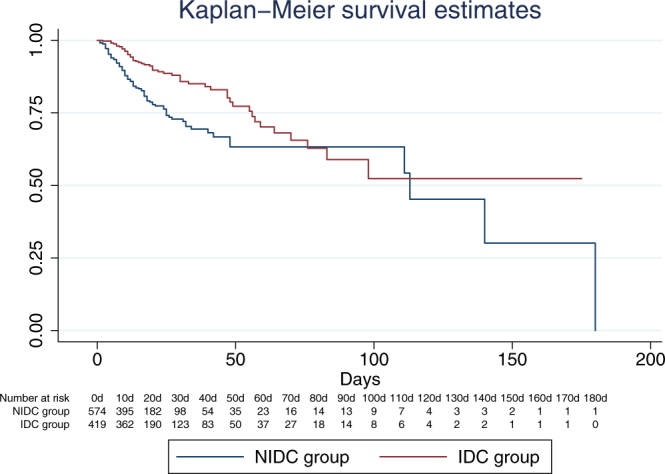
The KM-survival curve of the IDC and NIDC groups. (**A**) KM analysis for survival in all of the 995 patients with bloodstream infection according to ID consultation. The red line represents the IDC group (n = 421) and the blue line represents the NIDC group (n = 574). The survival estimates between the two groups is statistically significant (*P* < 0.05) using the log-rank test. Numbers of patients at risk in both groups at 10-day intervals are listed.

Furthermore, the KM-survival curve also indicated that the IDC group had advantages in survival over the NIDC group as shown in Fig. 1. The survival difference was statistically significant using the log-rank test (χ^2^ = 946.95, *P* < 0.05). In construction of this model, we also tried to test the interaction between ID consultation and age and that between ID consultation and organism type. However, the interactions were not statistically significant and therefore were not included into further investigations.

We also attempted to analyze the impact of ID consultation on the survival among cases with bloodstream infection due to different pathogens. Pathogens were divided into three subgroups as Gram-positive bacteria (seen in 231 patients), Gram-negative bacteria (in 677 patients) and fungi (in 87 patients). We used COX modeling to evaluate the impact of multi-variables on the hazard of death. ID consultation was still a protective factor among patients with bloodstream infection due to either Gram-positive (HR, 0.551; *P* < 0.05) or Gram-negative (HR, 0.331; *P* < 0.05) bacteria through two independent models analyzing Gram-positive and Gram-negative bacteria independently. However, the COX-PH model to evaluate the impact of multi-variables on the survival for bloodstream infection due to fungi failed to be constructed due to the small sample size.

### Multiple ID consultation and three-group of IDC COX modeling

Multiple ID consultation accounted for 52.7% (222/421) of all ID consultations. To evaluate the impact of single and multiple ID consultation on outcomes, we divided the whole cohort in three, i.e. single IDC, multi-IDC and NIDC, groups. The brief characteristics of the three groups were outlined in Table S1 in the supplementary file. Then we used COX modeling to evaluate the impact of multiple factors on the hazard of death as shown in Table 4. In this three-group modeling, risk factors were identical to those identified in the two-group (IDC and NIDC) modeling (see above) The protective factors were source control for infection foci and appropriate definitive antimicrobial therapy, which were also identified in the two-group modeling. However, single IDC as a variable that was supposed to be the protective factor was excluded from the model because of no statistical significance, suggesting that the benefits of IDC may be due to multiple-IDC (HR = 0.51; *P* < 0.05) rather than single-IDC.

**Table 4:** HR values (the single IDC group/multi-IDC group/NIDC group) generated from the COX model^a,b^.

Variables	HR	*P* value	95% CI
Age	1.022	<0.001	1.013–1.031
Hematology department	4.843	0.032	1.146–20.465
Oncology department	3.102	0.002	1.526–6.303
Multi-IDC	0.505	0.002	0.328–0.779
Hypotensive shock	2.715	<0.001	1.893–3.896
Renal insufficiency within 24 h	1.962	0.001	1.324–2.907
Mechanical ventilation within 7 d	1.767	0.002	1.240–2.519
Source control for infection foci	0.496	<0.001	0.353–0.698
Appropriate definitive antimicrobial therapy	0.593	0.033	0.367–0.958

^a^HR values refer to death or predicted death.

^b^Abbreviations: HR, hazard ration; IDC, infectious disease consultation; NIDC, non-infectious disease consultation; CI, confidence interval.

## Discussion

ID consultation has been associated with improved adherence to standards of care and better outcomes in previous studies^1,2,11,21–24^, but its impact depends on many human and organizational factors^25^. The improvement in outcome among patients who received ID consultation is very likely multifactorial and it would therefore be difficult to discern the effect of ID consultation on outcome. Nonetheless, based on the COX modeling results in our study, the HR of ID consultation was <1, suggesting that ID consultation truly provided significant advantages in survival over the NIDC group. To further evaluate benefits of ID consultation, prospective multi-center studies are warranted.

In China, the physician has no obligations to follow up the patient after the consultation. However, considering the change of the patient’s condition, a single consultation was unlikely to be adequate for certain patients. It was therefore not surprise that single ID consultation was not a protective factor as identified in the present study. It may also explain why some previous studies^2,3,9^ did not find the benefit of ID consultation in decreasing the hazard of death as the consultation might have been conducted only once.

Almost all of previous studies on the impact of ID consultation focused only on bacteremia due to *S. aureus* and fungaemia due to *C. glabrata*. To our knowledge, the present study is the first to confirm that ID consultation led to improved outcomes among patients with bloodstream infection due to both Gram-positive and Gram-negative bacteria. It is also noteworthy that the present study has a relatively large sample size, which could enhance the precision of our estimates towards the benefits of ID consultations on outcomes. The present study therefore adds to a growing body of evidence, suggesting that ID consultation optimizes management and improves outcomes of patients with bloodstream infections. We therefore recommend that ID consultation should be mandatory for patients with bloodstream infection as suggested previously^10^.

The present study had several limitations. First, the study used retrospective data with inevitable bias. The virulence and sequence types of pathogens, which may have an impact on the outcomes of patients, were not determined in this study due to its retrospective nature. Second, although the sample size was relative large, the study was performed at a single center and therefore the results may not be generalizable outside of this patient population. Third, ID consultation was most likely to be non-random but rather based on patients’ clinical conditions. ID consultation was therefore most likely to be conducted for patients with more severe conditions, which would compromise the positive effect of ID consultation. To minimize this selection bias, we adjusted for baseline characteristics using COX modeling in our analysis. Fourth, all-cause death rather than death due to bloodstream infection was used as the outcome as the exact reason of death was difficult to be determined in light of the low autopsy rate in China. Fifth, we did not measure the difference in cost between the IDC and NIDC groups. Sixth, in this study, several treatment measures such as foci removal had their own start time point, which were usually different from the start of observation, and may therefore introduce confounding effects into the model. Nonetheless, we introduced these covariates as time-fixed covariates, since the proportional presumption for these covariates was not rejected statistically. Last, we defined the infection site according to clinical suspicion to reflect the clinical practice rather than using standard criteria such as those in literature^26^.

In conclusion, patients with bloodstream infection that received ID consultation were significantly more likely to receive improved clinical management and were significantly less likely to die in hospitalization. For patients with bloodstream infection in China, ID consultation led to more survivals for both of those due to Gram-positive and Gram-negative bacteria, while multiple ID consultations rather than a single consultation reduced the hazard of death. ID consultation and follow up by ID physicians should be integrated in the clinical management for patients with bloodstream infection in developing countries like China.

## Electronic supplementary material

Table S1
