# Supplementary material for: Impact of Infectious Disease Consultation on Clinical Management and Outcome of Patients with Bloodstream Infection: a Retrospective Cohort Study
Source: Sci Rep. 2017 Oct 10;7:12898. doi: 10.1038/s41598-017-13055-2 (PMC5635120; doi:10.1038/s41598-017-13055-2)
Supplement: Supplementary file 1 — Table S1 [file 41598_2017_13055_MOESM1_ESM.pdf]

Impact of Infectious Disease Consultation on Clinical Management and Outcome of Patients with Bloodstream Infection: a Retrospective Cohort Study

Guangmin Tang[1], Liang Huang[1], Zhiyong Zong[1, 2§]

[1]Center of Infectious Diseases, West China Hospital, Sichuan University, Chengdu, China.

[2]Department of Infection Control, West China Hospital, Sichuan University, Chengdu, China.

Supplementary file

Table S1. Patients' brief baseline and clinical characteristics (single IDC/multi-IDC/NIDC)[a]

Characteristics	Single IDC N=199	Multi-IDC N=222	NIDC N=574	*P* value
Age (median)	51.9	52.9	55.5	.017
Male	120	127	372	.115
Hypotensive shock (within 24 h)	37	31	141	.002
Renal insufficiency (within 24 h)	22	11	83	.001
Mechanical ventilation with 7d	31	37	169	<.001
Malignance	72	53	164	.021
Deep vein catheterization	65	78	277	<.001
Pathogens				
Gram-positive bacteria	51	55	125	.435
Gram-negative bacteria	133	146	398	.564
Fungi	15	21	51	.79
Source control for infection foci	94	91	292	.043
Appropriate empirical antimicrobial therapy	137	190	385	<.001
Appropriate definitive antimicrobial therapy	185	215	503	<.001
Ward				
ICU	21	17	142	<.001
Internal Medicine (except Hematology and Oncology)	43	43	130	<.001
Surgical	85	66	170	.02
Emergence	24	53	61	<.001
Hematology	2	0	53	<.001
Oncology	23	11	14	<.001
Dermatology	1	1	1	.385
Ophthalmology	1	0	3	.65
Main underlying disease				
Action inconvenience, e.g. paralysis	16	19	44	.003
Chronic pulmonary disease	3	0	31	<.001
Chronic hepatic and biliary system disease	52	40	155	.025
Hematologic neoplasm	7	4	58	<.001
Vascular diseases e.g. aortic dissection	1	3	1	.071
Chronic renal insufficiency	21	4	47	<.001
Skin diseases	2	1	1	<.001
Connective tissue disease	2	2	8	.924
Diabetes	1	25	43	<.001
Burn or electric injury	0	4	9	.163
Urinary tract diseases except chronic renal insufficiency	16	8	20	.033
Chronic heart disease	4	15	18	.017

Dialysis	14	55	61	<.001
Immune suppression	44	21	99	.0956
Granulocytopenia	9	7	60	<.001
Parenteral nutrition	35	36	106	<.001
Complicated by infective endocarditis	2	8	11	.3295
Organ/space foci	115	145	362	.9833

[a]Abbreviations: IDC, infectious disease consultation; multi-IDC, multiple infectious disease consultations; NIDC, non-infectious disease consultation.
